# Surgical Outcomes in Pituitary Adenomas in a Resource-Limited Tertiary Government Hospital: A Five-Year Retrospective Cohort Study

**DOI:** 10.7759/cureus.109897

**Published:** 2026-05-29

**Authors:** Lester Ron S Bustamante, Jaime E Rama, Miguel Nicolai T Victorino

**Affiliations:** 1 Department of Neurosurgery, Vicente Sotto Memorial Medical Center, Cebu City, PHL

**Keywords:** cerebrospinal fluid rhinorrhea, diabetes insipidus, endoscopic transsphenoidal surgery, open transcranial surgery, pituitary adenoma, surgical outcomes

## Abstract

Introduction

Pituitary adenomas are among the most commonly encountered intracranial neoplasms requiring surgical intervention. Although endoscopic endonasal transsphenoidal surgery has become the preferred minimally invasive approach, open transcranial surgery continues to be indicated for selected invasive or anatomically complex lesions. Comparative outcome data from resource-limited tertiary institutions are still scarce. Therefore, this study aimed to evaluate the clinico-demographic characteristics and perioperative outcomes of patients with pituitary adenoma who underwent open or endoscopic pituitary surgery at a tertiary government hospital over five years.

Methods

A retrospective cohort descriptive-analytic study was conducted involving patients diagnosed with pituitary adenoma who underwent either open transcranial surgery or endoscopic transnasal transsphenoidal surgery at Vicente Sotto Memorial Medical Center from January 2018 to December 2022. Demographic characteristics, presenting symptoms, endocrine profiles, perioperative complications, mortality, length of hospital stay, and discharge outcomes were reviewed. Comparative analyses between surgical groups were performed using Fisher’s exact test and Mann-Whitney U test, with statistical significance set at p < 0.05.

Results

A total of 67 patients were included, of whom 18 (26.9%) underwent open surgery, and 49 (73.1%) underwent endoscopic surgery. Patients who underwent open surgery were older than those managed endoscopically (50.83 ± 11.26 years vs. 46.75 ± 12.11 years). Visual symptoms predominated in both groups, particularly blurring of vision and bitemporal hemianopsia. Non-functioning adenomas represented the majority of tumors in both cohorts (14; 77.8% vs. 40; 81.6%). Mean prolactin levels were markedly higher in the open surgery group (194.38 ± 633.28 ng/mL vs. 54.62 ± 103.17 ng/mL). The endoscopic cohort demonstrated a shorter mean hospital stay (21.41 ± 15.87 days vs. 23.67 ± 17.62 days), although the difference was not statistically significant (p = 0.512). Diabetes insipidus was the most common postoperative complication in both groups, occurring more frequently following open surgery (n = 5; 27.8% vs. n = 8; 16.3%). Cerebrospinal fluid rhinorrhea occurred exclusively in the endoscopic cohort (n = 3; 6.1%), whereas anemia and cavernous sinus injury were observed only in the open surgery group. Mortality rates were higher in the open surgery cohort (n = 2; 11.1% vs. n = 2; 4.1%), although no statistically significant differences were identified in complication rates, mortality, or discharge disposition between approaches. Most patients demonstrated postoperative improvement or recovery, particularly in the endoscopic cohort (n = 45; 91.8%).

Conclusions

Both open and endoscopic surgical approaches demonstrated favorable clinical outcomes in the management of pituitary adenomas. Endoscopic surgery was associated with shorter hospitalization and high postoperative recovery rates, supporting its role as the preferred minimally invasive approach for appropriately selected patients, while open surgery remained essential for complex lesions. These findings highlight that safe and effective pituitary surgery can be achieved in a tertiary government hospital within a resource-limited setting.

## Introduction

Pituitary adenomas, now referred to as pituitary neuroendocrine tumors (PitNETs), are benign epithelial neoplasms arising from the anterior pituitary gland and account for approximately 10% to 20% of all intracranial tumors, with an estimated prevalence of approximately one per 1,000 individuals in the general population [1-3]. Despite their benign histopathology, these tumors may cause significant neurologic and endocrinologic morbidity due to hormonal hypersecretion, hypopituitarism, and compression of adjacent neurovascular structures [4].

Pituitary adenomas are classified according to size and hormonal activity. Tumors measuring less than 10 mm are classified as microadenomas, while those greater than 10 mm are considered macroadenomas; giant adenomas measure more than 40 mm [5-10]. Functional adenomas secrete excess hormones, leading to clinical syndromes such as acromegaly, Cushing disease, and hyperprolactinemia, whereas non-functioning adenomas commonly present secondary to mass effect and compression of the optic apparatus [4,8-10]. Epidemiologic studies have shown that non-functioning adenomas comprise the majority of cases, followed by prolactinomas and growth hormone-secreting tumors [11].

Clinical manifestations vary depending on tumor size, invasiveness, and endocrine activity. Visual impairment remains the most common presenting symptom in macroadenomas due to suprasellar extension and compression of the optic chiasm, occurring in approximately 40-60% of patients [12-14]. Other manifestations include headache, cranial neuropathies, endocrine dysfunction, infertility, galactorrhea, acromegalic features, and symptoms of cortisol excess or deficiency [11-13]. Diagnosis relies on neuroimaging and biochemical evaluation. MRI with gadolinium remains the gold standard for assessing tumor extent and cavernous sinus involvement, while endocrine workup typically includes serum prolactin, cortisol, thyroid function tests, gonadotropins, growth hormone, and insulin-like growth factor-1 levels [8,9,12-15].

Management of pituitary adenomas requires a multidisciplinary approach involving neurosurgeons, endocrinologists, ophthalmologists, and neuroradiologists [8-10]. Medical therapy remains the first-line treatment for prolactinomas, particularly dopamine agonists such as cabergoline and bromocriptine [16-18]. However, surgery remains the cornerstone of treatment for symptomatic macroadenomas, tumors causing visual compromise, pituitary apoplexy, hormonally active tumors refractory to medical management, and lesions with significant mass effect [2,8,9,12]. The endoscopic endonasal transsphenoidal approach has become increasingly preferred because of its improved visualization, reduced morbidity, and favorable postoperative outcomes, although transcranial approaches remain necessary for select giant or invasive lesions [2].

Despite advances in surgical techniques, pituitary surgery remains associated with important complications, including cerebrospinal fluid leak, meningitis, diabetes insipidus, intracranial hemorrhage, visual deterioration, and hypopituitarism [19]. Larger and more invasive adenomas are associated with higher postoperative morbidity. Nevertheless, refinements in skull base reconstruction, particularly the use of vascularized nasoseptal flaps, have substantially reduced postoperative cerebrospinal fluid leak rates [20].

Locally, a study involving 71 patients with pituitary adenoma reported visual deficits in 80% of patients, headache in 49%, and diminished pituitary function in 45% [7]. Non-functioning adenomas were the predominant subtype, while diabetes insipidus was the most commonly encountered postoperative complication [7]. As the leading Department of Health-designated Brain and Spine Center in the Visayas, Vicente Sotto Memorial Medical Center manages a considerable number of patients with pituitary adenomas requiring surgical intervention. However, there is limited local data evaluating the demographic characteristics, clinical profile, treatment patterns, complications, and outcomes of patients undergoing open and endoscopic pituitary surgery in a tertiary government institution. This study aims to address these gaps and contribute local evidence that may guide surgical practice and patient care. The general objective was to determine the clinical profile and surgical outcomes of patients diagnosed with pituitary adenoma who underwent pituitary surgery at Vicente Sotto Memorial Medical Center from January 2018 to December 2022.

Specifically, the study aimed to determine the demographic profile of patients with pituitary adenoma in terms of age. Additionally, the study aimed to characterize the clinical profile of patients according to chief complaints, presenting signs and symptoms, and hormonal activity, including growth hormone-secreting adenomas, prolactinomas, ACTH-secreting adenomas, cortisol-secreting adenomas, thyroid-stimulating hormone-secreting adenomas, and non-functioning adenomas. Physical examination findings such as temporal hemianopsia, bitemporal hemianopsia, bitemporal quadrantanopsia, galactorrhea, light perception, and absence of light perception were also evaluated. Furthermore, diagnostic parameters including cortisol, prolactin, follicle-stimulating hormone, luteinizing hormone, thyroid-stimulating hormone, free thyroxine, free triiodothyronine, and fasting blood sugar levels were reviewed. Finally, the study described surgical treatment patterns involving open/transcranial surgery and endoscopic transnasal transsphenoidal surgery, as well as determined the complications and outcomes associated with each surgical approach.

## Materials and methods

Study design and setting

This study employed a retrospective cohort descriptive-analytic design and was conducted at Vicente Sotto Memorial Medical Center. Medical records of patients diagnosed with pituitary adenoma who underwent surgical intervention from January 2018 to December 2022 were reviewed.

Population

Inclusion Criteria

Adult patients diagnosed with pituitary adenoma who underwent either open/transcranial surgery or endoscopic transnasal transsphenoidal surgery at Vicente Sotto Memorial Medical Center from January 2018 to December 2022 were included in the study. Patients were included if their medical records contained sufficient demographic, clinical, operative, and perioperative data for analysis.

Exclusion Criteria

Patients managed non-surgically, those with insufficient medical records for perioperative outcome assessment, and those diagnosed with sellar or parasellar lesions other than pituitary adenoma were excluded from the study.

Statistical analysis

Data were entered and analyzed using RStudio version 4.2.0. Continuous variables were presented as mean ± standard deviation (SD) and compared using the Mann-Whitney U test due to non-normality and the relatively small sample size. Categorical variables were expressed as frequencies and percentages and analyzed using Fisher’s exact test because of small expected cell counts. Statistical significance was set at p < 0.05. Owing to the retrospective design, relatively small sample size, and likely differences in tumor complexity across surgical cohorts (Knosp grade, suprasellar extension, and cavernous sinus invasion were not consistently available), multivariable adjustment for potential confounders was not performed. Therefore, this study was descriptive and exploratory in nature and did not imply equivalence or a similar safety profile.

## Results

Table 1 presents the clinico-demographic characteristics of 67 patients who underwent surgical management for pituitary adenoma. Of these, 18 (26.9%) underwent open surgery, and 49 (73.1%) underwent endoscopic surgery. Patients who underwent open surgery were older, with a mean age of 50.83 ± 11.26 years, compared to 46.75 ± 12.11 years in those who had endoscopic surgery. A male predominance was observed in both groups, comprising 13 patients (72.2%) in the open surgery cohort and 29 (59.2%) in the endoscopic cohort.

**Table 1:** Clinico-demographic profiles of surgical patients SD: standard deviation; FSH: follicle-stimulating hormone; LH: luteinizing hormone; TSH: thyroid-stimulating hormone; FT4: free thyroxine; FT3: free triiodothyronine; FBS: fasting blood sugar

Variables	Open surgery (n = 18)	Endoscopic surgery (n = 49)
Age (years), mean ± SD	50.83 ± 11.26	46.75 ± 12.11
Sex, n (%)		
Male	13 (72.2)	29 (59.2)
Female	5 (27.8)	20 (40.8)
Chief complaint/presentation, n (%)		
Headache	4 (22.2)	12 (24.5)
Blurring of vision	14 (77.8)	36 (73.5)
Body malaise	1 (5.6)	0
Loss of vision	0	2 (4.1)
Seizure	1 (5.6)	1 (2.0)
Stiff neck	0	1 (2.0)
Functional, n (%)		
Cortisol-secreting adenoma	0	1 (2.0)
Cushing disease	0	1 (2.0)
Hypercortisolemia	0	1 (2.0)
Prolactinoma	3 (16.7)	1 (2.0)
Non-functional, n (%)	14 (77.8)	40 (81.6)
Physical examination, n (%)		
Temporal hemianopsia	0	1 (2.0)
Bitemporal hemianopsia	10 (55.6)	26 (53.1)
Bitemporal quadrantanopia	0	1 (2.0)
Galactorrhea	0	1 (2.0)
Light perception	1 (5.6)	1 (2.0)
No light perception	2 (11.1)	7 (14.3)
Diagnostics, mean ± SD		
Cortisol (ug/dl)	7.68 ± 8.87	13.47 ± 21.39
Prolactin (ng/ml)	194.38 ± 633.28	54.62 ± 103.17
FSH (mIU/ml)	7.64 ± 10.43	9.68 ± 15.13
LH (mIU/ml)	2.58 ± 3.68	4.29 ± 5.38
TSH (uIU/ml)	1.49 ± 0.94	1.59 ± 2.48
FT4 (ng/dl)	2.15 ± 3.22	1.54 ± 2.1
FT3 (pg/ml)	2.34 ± 2.4	2.11 ± 0.79
FBS (mg/dl)	130.71 ± 84.8	101.53 ± 19.72

Blurring of vision was the most common primary complaint in both surgical groups, occurring in 14 patients (77.8%) who underwent open surgery and in 36 patients (73.5%) who underwent endoscopic surgery. Headaches were the second most frequent symptom, reported in four patients (22.2%) in the open group and 12 patients (24.5%) in the endoscopic group. Other less common complaints included seizure, body malaise, stiff neck, and loss of vision.

Regarding tumor functionality, non-functioning adenomas comprised the majority of cases in both groups, representing 14 patients (77.8%) who underwent open surgery and 40 (81.6%) who underwent endoscopic surgery. Among functioning adenomas, prolactinoma was the most frequently encountered subtype in the open surgery cohort, accounting for three cases (16.7%). In contrast, isolated cases of cortisol-secreting adenoma, Cushing disease, and hypercortisolemia were observed in the endoscopic group.

On physical examination, bitemporal hemianopsia was the predominant finding in both groups, present in 10 patients (55.6%) undergoing open surgery and 26 patients (53.1%) undergoing endoscopic surgery. No light perception was documented in two (11.1%) and seven (14.3%) patients in the open and endoscopic cohorts, respectively. Other examination findings, including temporal hemianopsia, bitemporal quadrantanopia, galactorrhea, and light perception deficits, were infrequently observed.

Diagnostic hormonal evaluation demonstrated wide variability in endocrine parameters. Mean cortisol levels were higher in the endoscopic group (13.47 ± 21.39 µg/dL) compared to the open surgery group (7.68 ± 8.87 µg/dL). Likewise, mean prolactin levels were elevated in both cohorts, particularly among patients who underwent open surgery (194.38 ± 633.28 ng/mL versus 54.62 ± 103.17 ng/mL). Mean fasting blood sugar levels were also higher in the open surgery group (130.71 ± 84.8 mg/dL) compared with the endoscopic cohort (101.53 ± 19.72 mg/dL).

Table 2 demonstrates that the mean length of hospital stay was comparable between the two surgical approaches, with patients who underwent open surgery having a mean hospital stay of 23.67 ± 17.62 days, compared with 21.41 ± 15.87 days among patients who underwent endoscopic surgery. No significant difference was detected between the groups (p = 0.512).

**Table 2:** Comparison of perioperative outcomes between open and endoscopic surgical approaches ^a^Fisher's Exact test. ^b^Mann-Whitney U test P-value < 0.05 = statistically significant SD: standard deviation; CSF: cerebrospinal fluid

Outcomes	Open surgery (n = 18)	Endoscopic surgery (n = 49)	P-value
Length of hospital stay (days), mean ± SD	23.67 ± 17.62	21.41 ± 15.87	0.512^b^
Perioperative complications, n (%)			
Diabetes insipidus	5 (27.8)	8 (16.3)	0.319^a^
CSF rhinorrhea	0	3 (6.1)	0.556^a^
Hyponatremia	3 (16.7)	4 (8.2)	0.375^a^
Hypokalemia	1 (5.6)	1 (2.0)	0.468^a^
Postoperative hematoma	1 (5.6)	2 (4.1)	1.000^a^
Hospital-acquired pneumonia (HAP)	2 (11.1)	2 (4.1)	0.298^a^
Postoperative hemorrhage	1 (5.6)	1 (2.0)	0.474^a^
Anemia	1 (5.6)	0	0.272^a^
Cavernous sinus injury	1 (5.6)	0	0.272^a^
Mortality, n (%)	2 (11.1)	2 (4.1)	0.298^a^
Cause of mortality, n			
Brain herniation	1	1	—
Pituitary apoplexy	0	1	—
Postoperative hemorrhage	0	1	—
Discharge disposition, n (%)			
Improved/recovered	16 (88.9)	45 (91.8)	0.661^a^
Home against medical advice (HAMA)	0	2 (4.1)	1.000^a^

Diabetes insipidus was the most common perioperative complication in both cohorts, occurring in five patients (27.8%) who underwent open surgery and eight patients (16.3%) who underwent endoscopic surgery. Cerebrospinal fluid rhinorrhea was observed exclusively in the endoscopic group, occurring in three patients (6.1%). Hyponatremia occurred in three patients (16.7%) in the open surgery cohort and four (8.2%) in the endoscopic cohort. Other complications, including hypokalemia, postoperative hematoma, hospital-acquired pneumonia, postoperative hemorrhage, anemia, and cavernous sinus injury, occurred infrequently in both groups. No statistically significant differences were identified in perioperative complication rates between open and endoscopic surgery.

Mortality was observed in two patients (11.1%) who underwent open surgery and in two (4.1%) who underwent endoscopic surgery, although this difference did not reach statistical significance (p = 0.298). Causes of mortality included brain herniation, pituitary apoplexy, and postoperative hemorrhage.

Regarding discharge disposition, most patients demonstrated clinical improvement or recovery, defined as symptomatic relief or clinical stability at discharge, accounting for 16 (88.9%) of the open surgery cohort and 45 (91.8%) of the endoscopic surgery cohort. Two patients (4.1%) in the endoscopic group were discharged against medical advice. There was no significant difference in discharge outcomes between the two surgical approaches (p = 0.661).

## Discussion

This study evaluated the clinico-demographic characteristics, treatment patterns, perioperative complications, and outcomes of patients with pituitary adenoma who underwent surgical intervention at Vicente Sotto Memorial Medical Center from 2018 to 2022. Consistent with existing literature, pituitary adenomas in this cohort predominantly affected middle-aged adults and were more common in males [1-3]. The mean age of patients in both surgical groups was within the fifth decade of life, consistent with reports from international epidemiologic studies showing a peak incidence among middle-aged populations [11].

Visual disturbance, particularly blurring of vision, was the predominant presenting complaint in both surgical groups. Similarly, bitemporal hemianopsia was the most frequent physical examination finding. These findings are congruent with prior studies demonstrating that visual impairment secondary to optic chiasm compression remains the most common clinical manifestation of pituitary macroadenomas [13,14]. The high prevalence of visual symptoms in this study may reflect delayed consultation and advanced tumor size at presentation, a pattern commonly encountered in developing healthcare settings. Headache was also a common presenting symptom, consistent with prior reports attributing headache to sellar expansion and increased intrasellar pressure [13,14].

Non-functioning adenomas comprised the majority of tumors in both cohorts. This finding is consistent with local and international studies showing that non-functioning pituitary adenomas represent the most prevalent subtype encountered in surgical practice [7,11]. The predominance of non-functioning adenomas may explain the high frequency of visual deficits observed in this cohort, as these tumors often attain considerable size before detection due to the absence of hormonal syndromes [8-10].

Endocrine laboratory findings demonstrated substantial variability among patients, particularly in prolactin and cortisol levels. Elevated prolactin levels observed in both cohorts may be attributable either to prolactin-secreting adenomas or to the stalk effect secondary to compression of the pituitary stalk by large macroadenomas [8,9,13,15,16]. Likewise, the wide standard deviations in hormonal parameters likely reflect the heterogeneity of tumor functionality and the diverse endocrinologic presentations of pituitary adenomas.

The endoscopic transnasal transsphenoidal approach was the predominant surgical technique used at the institution. This finding reflects the evolving global preference for endoscopic skull base surgery, driven by improved visualization, panoramic access, reduced sinonasal morbidity, and favorable postoperative outcomes [2]. Nevertheless, open or transcranial surgery continued to play an important role in selected patients, particularly those with large or invasive tumors not amenable to standard transsphenoidal access.

With respect to perioperative outcomes, no statistically significant differences were observed between open and endoscopic surgery in terms of hospital stay, complications, mortality, or discharge disposition. Although not statistically significant, diabetes insipidus was the most common complication in both groups and occurred more frequently following open surgery. This finding is consistent with previous literature identifying diabetes insipidus as one of the most frequent postoperative endocrine complications following pituitary surgery due to manipulation of the hypothalamic-pituitary axis [7].

Cerebrospinal fluid rhinorrhea occurred exclusively in the endoscopic cohort, although the incidence remained low. This finding may reflect the increased exposure and wider access to the skull base afforded by endoscopic surgery. However, advances in reconstructive techniques, including vascularized nasoseptal flaps, have significantly reduced postoperative cerebrospinal fluid leak rates in contemporary practice. Other complications, such as postoperative hemorrhage, hyponatremia, hospital-acquired pneumonia, and cavernous sinus injury, occurred infrequently in both cohorts.

Mortality rates were higher in the open surgery group compared with the endoscopic cohort, although the difference was not statistically significant. Brain herniation, pituitary apoplexy, and postoperative hemorrhage accounted for the observed mortalities. The relatively higher mortality observed in open surgery patients may reflect greater tumor complexity, invasiveness, or disease severity necessitating transcranial approaches rather than the operative technique itself.

Most patients in both surgical cohorts demonstrated clinical improvement or recovery upon discharge, highlighting the overall effectiveness of surgical intervention in appropriately selected patients. Surgical decompression remains an important therapeutic modality for restoring visual function, relieving mass effect, and controlling hormonally active disease [2,8,9,13]. The outcomes between open and endoscopic surgery in this study suggest that both approaches remain viable and effective when tailored according to tumor characteristics and surgeon expertise.

Limitations

This study has several limitations. Firstly, its retrospective design relied on the completeness and accuracy of existing medical records, making it susceptible to missing documentation and information bias. The exclusion of patients with incomplete records may have also introduced selection bias. Second, the study was conducted in a single tertiary government institution, which may limit the generalizability of the findings to other centers or populations. Third, the relatively small sample size, particularly in the open surgery cohort, may have limited the statistical power to detect clinically meaningful differences between surgical approaches. Fourth, selection bias was inherent because patients who underwent open surgery likely had larger, more invasive, or anatomically complex tumors not amenable to standard endoscopic approaches. Radiologic parameters such as Knosp grade, suprasellar extension, and cavernous sinus invasion were not uniformly available and therefore could not be analyzed. Fifth, no multivariable adjustment for potential confounders was performed due to the retrospective design and limited sample size. Finally, long-term postoperative outcomes, including endocrine remission, visual recovery, tumor recurrence, progression-free survival, and quality of life, were not consistently available for analysis.

## Conclusions

Pituitary adenomas in this cohort predominantly presented with visual compromise and were largely non-functioning tumors requiring surgical decompression. Endoscopic transnasal transsphenoidal surgery demonstrated favorable perioperative outcomes and shorter hospital stays, underscoring its feasibility and acceptable perioperative safety in this resource-limited setting. Nevertheless, open transcranial surgery remained indispensable for anatomically complex or invasive lesions not amenable to standard endoscopic access. Although no statistically significant differences were identified between surgical approaches with respect to perioperative complications, mortality, or discharge outcomes, the interpretation of these findings should take into account the likely differences in tumor complexity and sample size, limiting any firm comparative inferences. Overall, the findings underscore the importance of individualized surgical planning based on tumor characteristics, disease extent, and operative expertise. This study further demonstrates that safe and effective pituitary surgery can be achieved within a tertiary government institution in a resource-limited setting, contributing meaningful local evidence to the evolving practice of skull base surgery in the Philippines.
